# Inhibitory interneuron circuits at cortical and spinal levels are associated with individual differences in corticomuscular coherence during isometric voluntary contraction

**DOI:** 10.1038/srep44417

**Published:** 2017-03-14

**Authors:** Ryosuke Matsuya, Junichi Ushiyama, Junichi Ushiba

**Affiliations:** 1 Graduate School of Science and Technology, Keio University, Kanagawa, Japan; 2 Faculty of Environment and Information Studies, Keio University, Kanagawa, Japan; 3 Department of Rehabilitation Medicine, Keio University School of Medicine, Tokyo, Japan; 4 Department of Biosciences and Informatics, Faculty of Science and Technology, Keio University, Kanagawa, Japan

## Abstract

Corticomuscular coherence (CMC) is an oscillatory synchronization of 15–35 Hz (β-band) between electroencephalogram (EEG) of the sensorimotor cortex and electromyogram of contracting muscles. Although we reported that the magnitude of CMC varies among individuals, the physiological mechanisms underlying this variation are still unclear. Here, we aimed to investigate the associations between CMC and intracortical inhibition (ICI) in the primary motor cortex (M1)/recurrent inhibition (RI) in the spinal cord, which probably affect oscillatory neural activities. Firstly, we quantified ICI from changes in motor-evoked potentials induced by paired-pulse transcranial magnetic stimulation in M1 during tonic isometric voluntary contraction of the first dorsal interosseous. ICI showed a significant, negative correlation with the strength of EEG β-oscillation, but not with the magnitude of CMC across individuals. Next, we quantified RI from changes in H-reflexes induced by paired-pulse electrical nerve stimulation to the posterior tibial nerve during isometric contraction of the soleus muscle. We observed a significant, positive correlation between RI and peak CMC across individuals. These results suggest that the local inhibitory interneuron networks in cortical and spinal levels are associated with the oscillatory activity in corticospinal loop.

Significant coherence between the sensorimotor cortex activity (measured by electroencephalogram (EEG) or magnetoencephalogram in humans, and local field potential in monkeys) and muscle activity, measured by electromyogram (EMG) of contracting muscles, was first reported ~20 years ago^1^,^2^. Corticomuscular coherence (CMC) has been considered to reflect the mutual interaction between the sensorimotor cortex and contracting muscles via descending motor pathways and ascending somatosensory pathways^3^,^4^,^5^,^6^,^7^. Recently, we have reported that the magnitude of CMC varies among individuals even in healthy young adults^8^,^9^,^10^. However, the physiological mechanisms underlying the inter-individual differences in CMC are still unclear. Although the inter-individual differences include some technical limitations for EEG/EMG, we believe that it is valuable to examine the physiological mechanisms behind inter-individual differences in CMC, since CMC is associated with insensible personal behaviour such as force steadiness^10^ and reaction time^11^.

Negative-feedback systems are known to generate oscillatory output^12^,^13^,^14^,^15^; this implies that inhibitory neural circuits are associated with CMC. A pharmacological study reported that 20 Hz oscillations in the sensorimotor cortex are partially produced by local cortical circuits relying on GABA_A_-mediated intracortical inhibition (ICI)^16^. Thus, we hypothesised that ICI is a factor of individual differences in cortical β-oscillation, and also in CMC if oscillatory descending drives are directly transmitted to the periphery. However, the oscillations can be modulated at the spinal level. Renshaw cells are known to regulate oscillations in muscle activity by preventing synchronization of spinal motoneuron activity^17^,^18^,^19^. Therefore, we formulated the second hypothesis that recurrent inhibition (RI) of Renshaw cells is a second factor of individual differences in CMC.

The present study aimed to test the two aforementioned hypotheses. Firstly, we examined the relationship between CMC and ICI using the paired-pulse transcranial magnetic stimulation (TMS) method among healthy participants. We measured the surface EMGs from the first dorsal interosseous (FDI) muscle in ICI experiments because motor-evoked potentials (MEPs) are detected from finger muscles in TMS. Secondly, we examined the relationship between CMC and RI using the paired-pulse H-reflex method among healthy participants. We measured the surface EMGs from the soleus (SOL) in RI experiments, because RI, which can be quantitated by H-reflex method^20^, has been mostly evaluated from SOL^21^,^22^. We integrated the results from the two experiments and evaluated cortical and spinal factors related to inter-individual differences in CMC.

## Results

### ICI and CMC during FDI contraction

**Figure 1:**
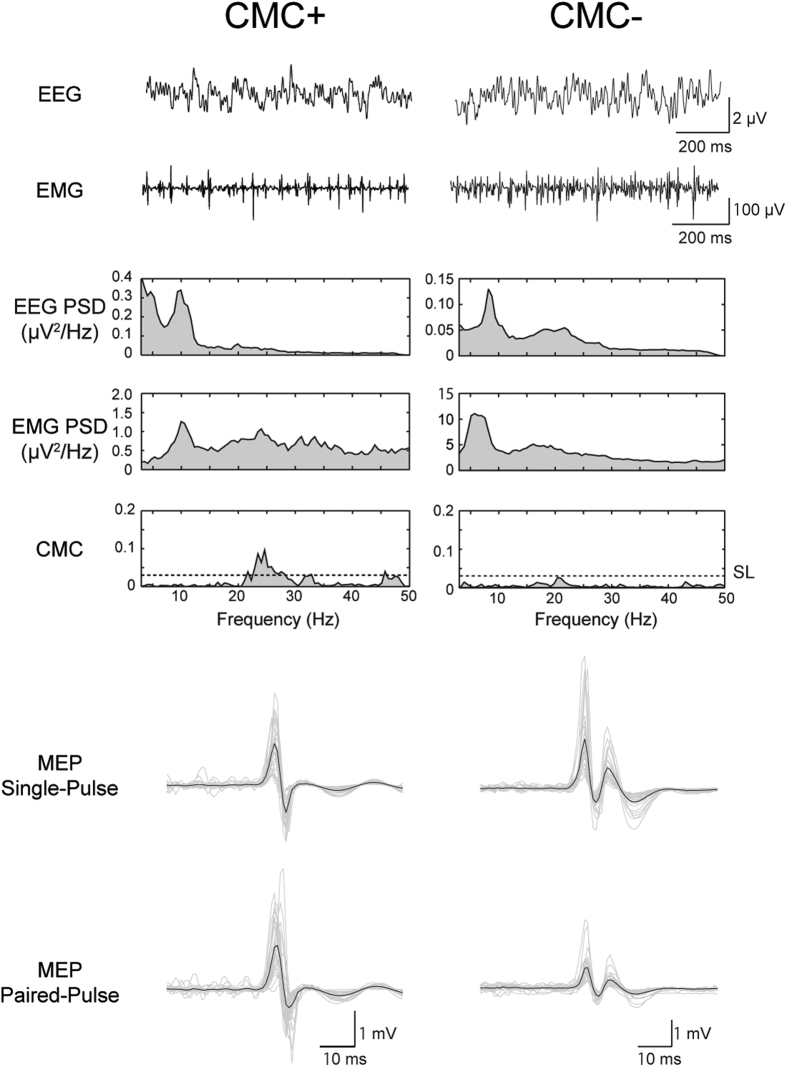
Representative examples of EEG/EMG data and motor-evoked potentials (MEPs) for a participant who showed significant CMC (CMC+) and a participant who did not (CMC−). Raw EEG signals, raw EMG signals, power spectral density functions (PSDs) for EEG and rectified EMG signals, corticomuscular coherence (CMC) spectra during isometric contraction of the first dorsal interosseous (FDI), and MEPs elicited by single-pulse and paired-pulse transcranial magnetic stimulation (TMS) are shown. In the CMC spectra, the estimated significance levels of coherence (SLs, 0.030) are shown as horizontal dotted lines. In MEPs, grey lines show the individual EMG responses of 30 trials, and black lines show averaged waves.

**Figure 2:**
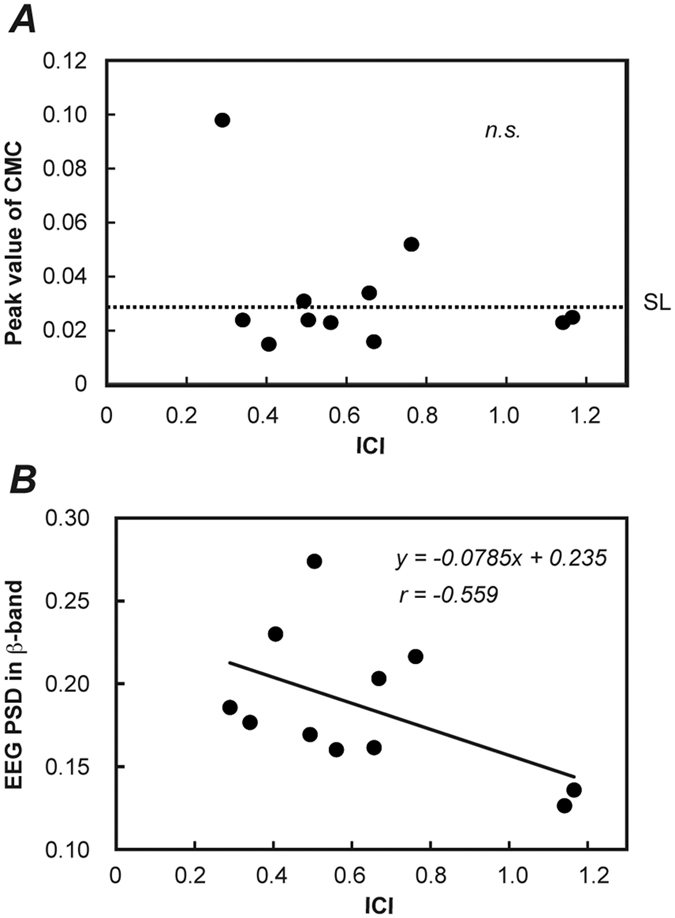
(**A**) The relationship between intracortical inhibition (ICI) and the peak value of CMC during isometric contraction of FDI across all participants. A horizontal dashed line shows the estimated SL (=0.030). There was no significant correlation between CMC and ICI (*p* = 0.197). (**B**) The relationship between ICI and EEG PSD in β-band across all participants. EEG PSD in β-band indicates the ratio of the sum of EEG-PSD within the β-band (15–30 Hz) to that of 3–50 Hz frequency range. There was a significant correlation between them (*r* = −0.559, *p* = 0.037). The solid line shows the estimated regression line.

We calculated CMC from the EEG/EMG data during the isometric contraction of FDI without TMS and observed that the magnitude of CMC differed among the present participants. We also calculated values of ICI from the MEPs during the contractions with TMS. Figure 1 shows raw EEG and EMG signals, EEG and rectified EMG-power spectrum densities (PSDs), CMC, and MEPs recorded from 2 representative participants showing significant CMC (CMC+) and non-significant CMC (CMC−). Grouped discharges were observed in raw EMG waves of the CMC + participant, and β-peak was remarkable in the rectified EMG-PSD of the CMC+ participant than in that of the CMC− participant. However, MEP reduction because of the paired-pulse method was observed more clearly in the CMC− participant than in the CMC+ participant. These comparisons between CMC+ and CMC− participants were in contrast with our first hypothesis that the stronger the ICI, the greater the CMC. No significant correlation was detected between the peak values of ICI and CMC across all participants (*p* = 0.197) (Fig. 2A). However, EEG β-PSD correlated significantly and negatively with ICI (Fig. 2B) (*r* = −0.559, *p* = 0.037) (i.e. the stronger the ICI, the more prominent the EEG β-oscillations). As shown in Fig. 1, the CMC− participant had a more distinct β-band power in EEG PSD than the CMC− participant, although β-oscillations were observed in raw EEG waves of both participants.

### RI and CMC during SOL contraction

**Figure 3:**
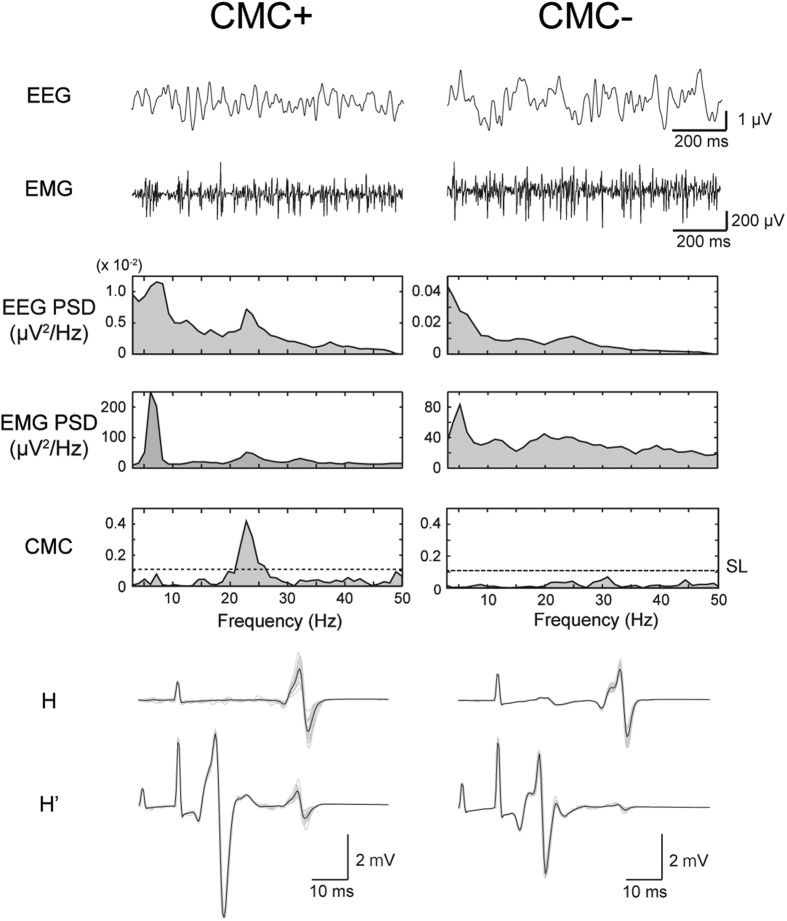
Representative examples of EEG/EMG data and H-reflexes for CMC+ and CMC−. Raw EEG signals, raw EMG signals, power spectral density functions (PSDs) for EEG and rectified EMG signals, CMC spectra during isometric contraction of the soleus (SOL), and H-reflexes elicited by single (H) and paired-pulse (H′) electrical nerve stimulation are shown. Data for CMC+ and CMC− are shown, respectively. In the CMC spectra, horizontal dashed lines show the estimated SL (=0.091). In H-reflexes, grey lines show individual EMG responses of 20 trials, and black lines show averaged waves.

**Figure 4:**
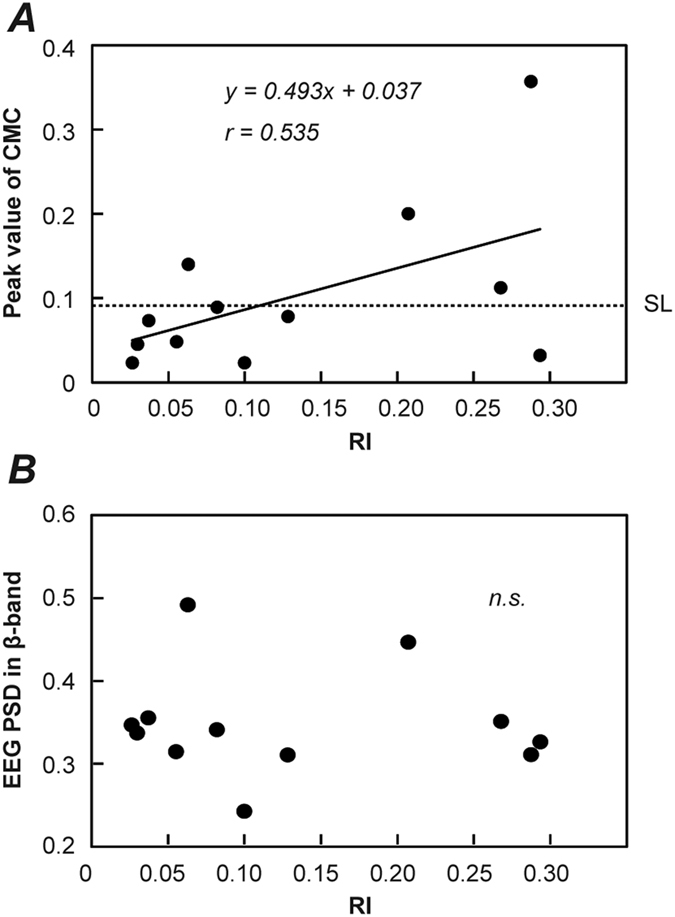
(**A**) The relationship between recurrent inhibition (RI) and the peak value of CMC during the isometric contraction of SOL across all participants. A horizontal dashed line shows the estimated SL (=0.091). A significant correlation was observed between RI and CMC (*r* = 0.663, *p* = 0.009). The solid line shows the estimated regression line. (**B**) The relationship between RI and EEG PSD in β-band across all participants. EEG PSD in β-band indicates the ratio of the sum of EEG-PSD within the β-band (15–30 Hz) to that of 3–50 Hz frequency range. There was no significant correlation between them (p = 0.270).

We calculated CMC from the EEG/EMG data during isometric contraction of SOL without electrical nerve stimulation and observed that the magnitude of CMC in SOL differed among the present participants. We also calculated values of RI from H-reflex amplitudes measured in RI experiment (EXP_RI_). Figure 3 shows the raw EEG and EMG signals, EEG and rectified EMG-PSDs, CMC, and H-reflexes recorded from 2 representative participants (they are different individuals from the representative participants shown in Fig. 1). As shown in Fig. 3, the CMC+ participant showed remarkable peaks at 22 Hz in EEG, rectified EMG and CMC, and oscillatory activities were observed in raw EEG/EMG in contrast with the CMC− participant. An amplitude reduction from H to H′ was much more prominent in the CMC− participant than in the CMC+ participant, a certain level of reduction was observed between both groups of participants. This difference between participants implies that RI is stronger in CMC− participants than in CMC+ participants. To confirm whether the correlation between CMC and RI was significant, we plotted the peak values of CMC and RI during contraction task for all participants (Fig. 4A). According to the difference between representative participants, there was a significant positive correlation between them (*r* = 0.663, *p* = 0.009) (i.e. the stronger the RI, the weaker the CMC). On the other hand, there was no significant correlation between RI and EEG β-PSD (*p* = 0.26) (Fig. 4B).

### Inter-individual correlation between CMCs recorded from FDI and SOL

**Figure 5:**
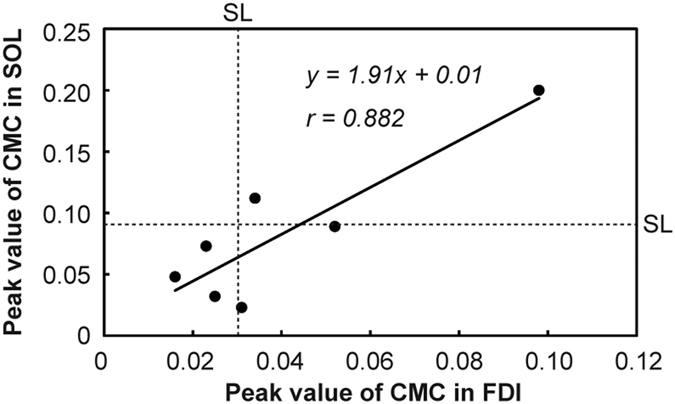
The relationship between peak values of CMC recorded from FDI and SOL. The horizontal and vertical dashed lines show the estimated SL for FDI (=0.030) and for SOL (=0.091), respectively. There was a significant positive correlation between them (*r* = 0.882, *p* = 0.004). The solid line shows the estimated regression line.

We plotted the association between peak values of CMC recorded from FDI and SOL for 7 individuals who participated in both ICI experiment (EXP_ICI_) and EXP_RI_. A significant positive correlation was observed between them (*r* = 0.882, *p* = 0.004) (Fig. 5).

## Discussion

Firstly, we demonstrated that there is a distinct variation in EEG β-PSD across participants, similar to our previous study including 100 participants^10^, and a significant negative correlation between the strength of ICI and EEG β-PSD (note that the stronger the ICI, the larger the EEG β-PSD) (Fig. 2B). The value of ICI measured by a paired-pulse TMS method represents the strength of inhibition of GABA_A_-mediated interneurons^23^. Thus, our results suggest that the greater the inhibition of GABA_A_-mediated interneurons, the more prominent the β-band-synchronized activities of neurons within the sensorimotor cortex. Oscillations occur by the reverberation around feedback loop with a conduction delay. An intracortical circuit was shown that generated oscillations as an emergent network property arising from their local circuit connectivity^15^,^24^,^25^,^26^. A modelling study showed that the inhibitory interneurons played a key role in determining the oscillation frequency and amplitude, such that increase in the inhibitory connections lead to increase in the strength of oscillations^15^. Concurrently, a pharmacological study reported that 20 Hz oscillations in the sensorimotor cortex were strengthened by administration of a drug enhancing GABA_A_-mediated inhibition^16^. In addition, a genetic study identified a significant linkage between β-oscillation in EEG and a set of GABA_A_ receptor genes^27^. Taken together, this indicates that the strength of intracortical inhibitory interneuron activities might be determined by genetic variation of GABA receptor, and this might be a factor determining the inter-individual differences in the magnitude of EEG β-oscillation.

However, as shown in Fig. 2B, the obtained correlation between ICI and EEG β-oscillation was not strong but moderate (*r* = −0.559; *p* = 0.037). Not only ICI, but also other neural activities might be associated with producing oscillatory neural activities in the sensorimotor cortex. Neurons in the primary motor cortex are known to exhibit an intrinsic tendency to fire rhythmically^28^,^29^. It has been suggested that cell populations tend to synchronize repetitive firing at rates close to β-band because the probability of firing increases at ~30 ms after the previous action potential. Furthermore, Roopun *et al*.^30^ reported that layer V pyramidal neurons have gap-junctional connections between their axons, which lead to strong electrical coupling in the absence of synaptic activity. Therefore, althou ICI should be a determinant of individual strength of the β-oscillation in the sensorimotor cortex during isometric contraction, there might be other neural factors associated with cortical oscillations.

Significant correlation was observed only between the value of ICI and EEG β-oscillation but not between ICI and magnitude of CMC among participants (Fig. 2A). β-band CMC is considered to be a bidirectional phenomenon including descending and ascending neural signal flow^3^,^6^,^7^. Thus, oscillation descending from sensorimotor cortex to muscles is not the sole determinant of the magnitude of CMC. Comparing the power spectra of representative individuals (Fig. 1), the CMC+ participant showed more prominent EMG PSD in β-band than the CMC− participant, in agreement with the positive correlation between CMC and EMG β-PSD among individuals reported in our previous study 10, the CMC− participant had more distinct β-PSD in EEG rather than the CMC + participant. It is difficult to consider that the cortical oscillation is simply transmitted to the muscle.

Next, we focused on the spinal modulation of oscillatory corticospinal loop activity. The spinal cord is not only a relay point between the motor cortex and muscles, but also a regulator of activation of spinal motoneurons by its neural circuits. It is known that RI produced by Renshaw cells regulates motoneuron excitability and stabilizes firing rate. Moreover, this negative feedback system has been reported as a mechanism for reduction of oscillatory muscle activation by preventing motoneuron synchronization^17^,^18^,^19^. Therefore, to determine whether the RI is associated with the individual magnitude of CMC, we performed EXP_RI_ using H-reflex method^20^.

The result from the RI experiment demonstrated a significant positive correlation between the strength of RI and the magnitude of CMC (Fig. 4A). This indicates that the greater the RI generated by spinal Renshaw cells, the weaker the magnitude of CMC. Recently, Williams and Baker^31^ reported that this inhibitory feedback plays a role as ‘neural filter’ and improves physiological tremor by reducing the magnitude of CMC at 10 and 20 Hz.

In addition, we considered the possibility that the spinal oscillation modulated by RI may influence cortical β-oscillation via ascending feedback. However, no significant correlation was detected between the RI and EEG β-oscillation across the individuals (Fig. 4B). This implies that some cortical-specific modifications have greater influences on the cortical oscillation than the effect of modulation by RI via ascending feedback. Thus, we suggest that the modulation by Renshaw cell activity works to weaken the oscillation derived from the cortex as ‘neural filter’, although the effect of modulation by RI may not be transmitted to a great extent; therefore, the strength of the RI was positively correlated with the magnitude of CMC.

In the ICI experiment, we recorded EMG from FDI; however, in the RI experiment, EMG was recorded from SOL. As we reported previously^9^, a remarkable inter-participant difference in CMC was observed from distal lower limb muscles than from those of upper limb. Therefore, we used mainly the TA or SOL for EEG-EMG assessments^10^,^11^,^32^. Moreover, H-reflex is usually recorded from SOL^21^,^22^, and in our experience, it is very difficult to record H-reflex from upper limb muscles in East Asian population. Therefore, we decided to record EMG from SOL in EXP_RI._ However, it is not easy to detect MEPs from lower limb muscles because representative area of the lower limb in the motor cortex is located deep in the longitudinal fissure. Thus, most of the ICI studies using TMS method would have used upper limb muscles to recorded MEPs^33^,^34^. Hence, we selected FDI that has been widely used to detect MEPs as a target muscle in EXP_ICI_.

While this difference in recorded muscles between two experiments was unavoidable because of the aforementioned technical limitations, one might claim that background neural networks vary between motor areas of different muscles. As shown in Fig. 5, we observed a strong significant correlation between peak values of CMC for these muscles, although the strength of CMC for FDI was much weaker than that for SOL. Thus, the inter-individual variations in CMC seem to be retained across muscles. This strong correlation led us to speculate that the tendency of cortical and/or spinal motoneurons to fire synchronously is partially common among skeletal muscles.

However, we should not discuss physiological mechanisms underlying “CMC variation among muscles” and “CMC variation among individuals” in a similar way. For example, the significant positive correlation between CMC and RI in the SOL suggests that the strength of RI is a factor of individual difference in CMC recorded from the SOL. However, CMC in FDI is weaker than that in the SOL, although it is generally assumed that RI is absent in intrinsic hand muscles^35^,^36^. Thus, we cannot explain the difference in the magnitude of CMC between FDI and SOL from the view point of RI at the spinal level. Difference in CMC across individuals and/or muscles would be enmeshed with physiological factors such as density of cortical projection^37^,^38^, or the strength of local inhibitory circuits such as ICI and RI and other historical factors such as development, aging, and frequency of muscle use.

**Figure 6:**
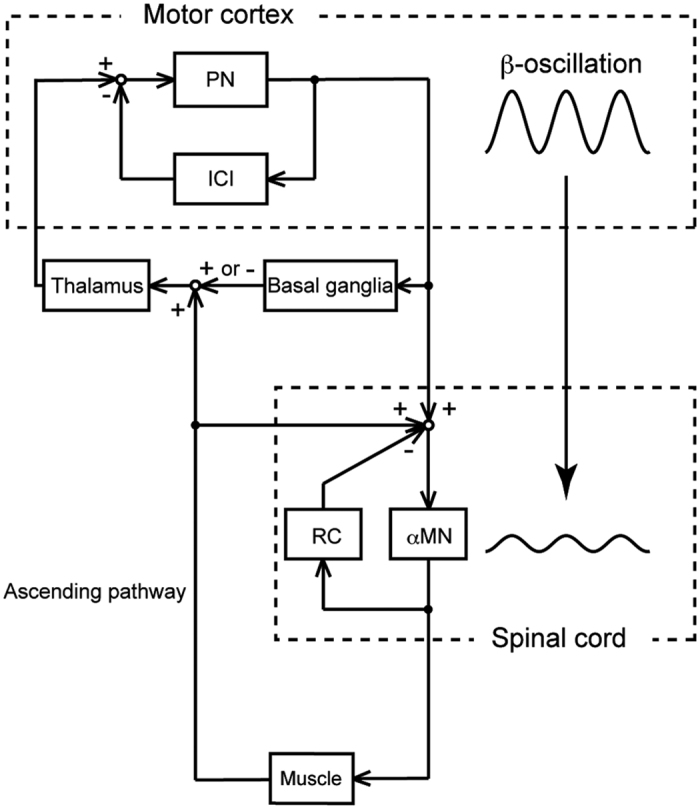
Model for the neural system associated with the development of CMC during voluntary isometric contraction. The present two main findings suggest the roles of ICI and RI for β-oscillation in the corticospinal pathway. At first, the negative feedback loop between pyramidal neurons (PN) and ICI generates β-oscillation. Next, RI of Renshaw cells (RCs) desynchronizes α-motoneurons (αMNs) firing and attenuates the amplitude of β-oscillation derived from the motor cortex.

EEG and EMG assessments also include technical limitations derived from non-physiological factors. For example, the electrical field depends on thickness of scalp and skull in case of EEG^39^,^40^,^41^,^42^, and spatial filter design of EMG is influenced by fat/skin tissues^43^ and electrode locations^44^,^45^. These factors influence the EEG/EMG amplitudes. However, the magnitude of coherence is known to reflect the constancy of the amplitude ratio and/or the phase difference between 2 signals throughout data. Thus, the influence of aforementioned factors is considered limited. Moreover, the present study succeeded in detecting significant correlations between the neural inhibitions and β-band oscillatory activities in the corticospinal pathway using electrophysiological methods and indicated that the inter-individual difference in CMC presumably derives from the physiological factors. Therefore, the finding that neural factors associated with β-band CMC were found physiologically is even more significant. These data demonstrate that individual magnitude of CMC is associated with inner physiological factors in both cortical and spinal levels. Taking the present two main findings on ICI and RI together, we suggest that the magnitude of CMC includes the effects of cortical and spinal inhibitory circuits, such as ICI and RI, on synchronous neural activities. Cortical inhibitory circuits should have a role to generate cortical β-oscillations. On the other hand, spinal inhibitory circuits presumably modulate synchronizations of spinal α-motoneurons (Fig. 6). Previous studies have regarded CMC not only as the phenomenon reflecting the descending information flow but also as the bidirectional interaction between sensorimotor cortex and contralateral muscles. The present study would provide additional speculations that CMC is associated not only with the neural loop including efferent and afferent pathways, but also the local loops at the cortical and spinal levels.

## Methods

The experiments were approved by the local ethics committee of the Faculty of Science and Technology, Keio University, Yokohama, Japan, and were conducted in accordance with the Declaration of Helsinki. All participants provided their informed consent for the study after receiving a detailed explanation of the purpose, potential benefits, and risks involved.

### Participants

In total, 16 healthy young adults (11 male and 5 female participants; aged 21–35 years) with no history of neurological disorders voluntarily participated in this study. Eleven participants (6 male and 5 female participants; aged 21–24 years) attended the ICI experiment (EXP_ICI_), and 12 participants (8 male and 4 female participants; aged 22–35 years) attended the RI experiment (EXP_RI_). Seven individuals (3 male and 4 female participants) participated in both of EXP_ICI_ and EXP_RI_.

### Recordings

EEG was recorded from the scalp region overlying the sensorimotor cortex using 5 Ag/AgCl electrodes of 10 mm in diameter, placed at areas representative of the muscles to be investigated in each experiment (i.e. C3 and 20 mm frontal, back, left, and right positions in EXP_ICI_; Cz and 4 surrounding positions in EXP_RI_, defined by the International 10–20 system). We placed the reference electrode for EEG at A1 (left earlobe) and the ground electrode on the forehead. EEG signals were derived using the spatial Laplacian filter^46^. Surface EMG was recorded from right FDI in EXP_ICI_ or from right SOL and tibialis anterior (TA) muscles in EXP_RI_. The bipolar Ag/AgCl electrodes were attached to the belly of each muscle with an inter-electrode distance of 20 mm.

In EXP_ICI_, EEG/EMG signals were amplified, band-pass filtered (EEG, 1–200 Hz; EMG, 10–500 Hz), and digitised at 1200 Hz using an EEG/EMG recording system (gUSB amp, Gugaer Technologies, Graz, Austria). The force signal was recorded with a strain gauge (DPM711B, Kyowa Electronic Instruments, Japan), and digitised at 1200 Hz by an analogue-to-digital converter (DAQCard-6062E; National Instruments Inc., Austin, TX, USA). In EXP_RI_, EEG signals were amplified, band-pass filtered (2–500 Hz), and digitised at 2400 Hz using the same recording system. EMG signals were amplified and band-pass filtered (20–1000 Hz) using a bio-signal recording system (Neuropack X1 MEB-2306; Nihon Kohden Corporation, Tokyo, Japan). Plantar flexion force was recorded with an ankle-dynamometer (MK-808052; ME incorporated, Nagano, Japan) and low-pass filtered at 50 Hz. EMG and force signals were digitised at 2400 Hz by an analogue-to-digital converter (NIUSB-6259 BNC, National Instruments Inc.). We originally designed measurement programs using MATLAB software (The MathWorks Inc., Natick, MA, USA).

### Stimulation

#### TMS

TMS was applied using a figure-eight shaped coil connected to the two Magstim 200 magnetic stimulators (Magstim, Whitland, UK). The optimal coil position where MEPs in FDI could be evoked with the lowest stimulus was marked with ink to ensure an exact repositioning of the coil throughout the experiment. The handle of the coil oriented backward so that the induced current was directed from posterior to anterior. At this position, the motor threshold (MT) intensity was defined as the lowest stimulator output intensity, capable of inducing an MEP of at least 50 μV peak-to-peak amplitude in relaxed muscles in at least half of the 10 trials. A sub-threshold conditioning stimulus (CS) was set at 80% of the MT, and a supra-threshold test stimulus (TS) was set at 120% of the MT^47^. Only TS was delivered for the single-pulse, and CS was applied through the same coil at 3 ms prior to TS for the paired-pulse^33^. Before the experiment, we confirmed that MEPs were not evoked by only CS during weak contractions.

#### H-reflex

H-reflex of the SOL was evoked by electrically stimulating the posterior tibial nerve. The cathode shaped as a half-ball (2 cm diameter; TF-98003, Unique Medical, Tokyo, Japan) was placed over the popliteal fossa. The anode Ag/AgCl electrode (2000 mm^2^; 019-768500, VIASYS Healthcare, Woking, UK) was placed immediately proximal to the patella. Two stimuli with different intensities (Stim1 and Stim2) as a 1 ms rectangular pulse were delivered by an electronic stimulation system (SEN-3301/SS-104J, Nihon Kohden, Tokyo, Japan). Stim1 was adjusted so that maximal amplitude of H-reflex was observed, and Stim2 was defined to elicit maximal M-wave (M_max_) followed by no H-reflex during the contraction (about 110% of the stimulus intensity at which we first elicited M_max_). Only Stim1 was provided for the single-pulse, while Stim1 and Stim2 were given together with an interval of 10 ms for the paired-pulse^20^. The preceding Stim1 not only elicits H-reflex, but also activates Renshaw cells which feedback inhibition to the same α-motoneuron pool. When Stim2 is applied just 10 ms after Stim1, the H-reflex discharge evoked by Stim1 collides an antidromic motor volley caused by the following Stim2. However, Ia inputs caused by Stim2 can activate the α-motoneurons, evoking the second H-reflex (H′). As this H′-reflex is affected by an inhibitory input from RI, which is activated by Stim1, its amplitude is smaller than the H-reflex evoked by single pulse method. Because of such mechanism, the H-reflex inhibited by RI can be detected by the paired-pulse H-reflex method.

### Experimental protocol

#### EXP_ICI_

Each participant was comfortably seated on a chair, and the right hand was supported by the splint with the strain gauge. Before the experiment, we measured force levels of the FDI during maximal voluntary contraction (MVC). Firstly, the participants performed isometric voluntary contraction tasks of the FDI at 5% of MVC without stimulation. The participants repeated FDI contraction for 15 s with a rest interval of 5 s for 35 times in total^48^. During this task, participants were given a visual feedback about the level of abduction force provided via a level meter on a computer screen positioned 0.5 m in front of them, and were instructed to maintain their exerted force with accuracy.

Next, TMS was applied over the hand area of the left primary motor cortex 60 times (single-pulse, 30 times; paired-pulse, 30 times) in a random order during the isometric contraction. The participants were instructed to perform 5% MVC abduction for 15 s and were provided the stimuli twice in a trial at unpredictable times within 5 s ± 500 ms and 13 s ± 500 ms after an onset of contraction. The task included a series of 30 trials of 15 s each with a rest interval of 5 s.

#### EXP_RI_

Each participant was comfortably seated on a chair with an ankle dynamometer. Before the experiment, we measured MVC of SOL. Firstly, participants performed isometric voluntary contraction task of SOL at 15% MVC for 60 s without stimulation. The participants were given the feedback of plantar flexion force level provided on the screen positioned 1.2 m front of them, and were instructed to keep their force levels.

Next, the stimulus was provided to the participants during the contraction. Before the measurement, we determined the intensity of Stim1 and Stim2 during the isometric contraction at 15% MVC. The participants repeated the contraction for 7 s with a rest interval of 15 s for 40 times in total, and were provided the stimulus (single-pulse or paired-pulse) at unpredictable times within 6 s ± 500 ms after an onset of the contraction (i.e. single-pulse, 20 times; paired-pulse, 20 times in total). After the experiment, the participants were instructed to perform additional contractions and were provided only S2, to obtain M_max_ responses.

### Data analysis

#### Coherence analysis

For the data of the no-stimulation tasks, we evaluated power spectral density using Welch’s method for EEG and rectified EMG signals and CMC. Before the following analyses, EEG signals were band-pass filtered (2–100 Hz), and Laplacian-filtered signals at C3 were calculated for EXPICI or at Cz for EXPRI. To detect the motor unit grouped discharge, we used the rectification for EMG. This pre-processing is necessary to extract the envelope of the modulation wave^49^,^50^,^51^,^52^. Raw EEG and rectified EMG signals were segmented into artefact-free epochs including 2048 data points. We obtained non-overlapped 245 epochs in total (7 epochs per contraction for 15 s) in EXP_ICI_ and 70 epochs from contraction for 60 s in EXP_RI_. Each epoch was convoluted with Hanning-window to reduce spectral leakage^2^,^53^,^54^. We determined correlations between EEG and rectified EMG using coherence function^55^ (see Appendix A), and estimated the 95% significant level (SL)^55^,^56^,^57^ (see Appendix B). We also determined the ratio of the sum of the PSD within the β-band (15–30 Hz) to that of 3–50 Hz frequency range for EEG-PSD, and these values were defined as EEG β-PSD.

#### ICI estimation

Firstly, we performed signals averaging for 30 EMG responses obtained from each of single-pulse and paired-pulse TMS methods, and defined peak-to-peak amplitudes of the averaged waves as MEPs. Next, to confirm that ICI was elicited by the present paired-pulse TMS method, we examined differences in MEP between single-pulse and paired-pulse methods, using Wilcoxon signed-rank test (single = 3.05 ± 1.98 mV, paired = 1.85 ± 1.40 mV, p = 0.008). The ratio of the conditioned MEP to the unconditioned MEP was determined as a measure of ICI. Accordingly, a smaller value indicated greater inhibition.

#### RI estimation

Firstly, as in ICI estimation, we provided signals averaging for 20 EMG responses obtained from each stimulation, and defined peak-to-peak amplitudes of the averaged waves as H or H′. Next, to confirm that RI was elicited by the present paired-pulse H-reflex method, we examined differences between H and H′ using Wilcoxon signed-rank test (single = 6.75 ± 2.99 mV, paired = 0.88 ± 0.72 mV, p = 0.002). The ratio of H′ to H was determined as a measure of RI, and smaller RI meant greater inhibition.

#### Statistical analyses

Coherence was normalized using the arc hyperbolic tangent transformation for statistical analyses^55^. To confirm whether there were significant correlations between ICI and the peak magnitude of CMC between ICI and EEG β-PSD between RI and the peak value of CMC and between RI and EEG β-PSD, Pearson correlation coefficients between these values were determined. All statistical analyses were performed using SPSS software (IBM SPSS Inc., Armonk, New York, USA).

## Additional Information

**How to cite this article:** Matsuya, R. *et al*. Inhibitory interneuron circuits at cortical and spinal levels are associated with individual differences in corticomuscular coherence during isometric voluntary contraction. *Sci. Rep.* **7**, 44417; doi: 10.1038/srep44417 (2017).

**Publisher's note:** Springer Nature remains neutral with regard to jurisdictional claims in published maps and institutional affiliations.

## Supplementary Material

Supplementary Appendix
