# Supplementary material for: Inhibitory interneuron circuits at cortical and spinal levels are associated with individual differences in corticomuscular coherence during isometric voluntary contraction
Source: Sci Rep. 2017 Mar 14;7:44417. doi: 10.1038/srep44417 (PMC5349562; doi:10.1038/srep44417)
Supplement: Supplementary Appendix [file srep44417-s1.pdf]

Supplementary Information of "Inhibitory interneuron circuits at cortical and spinal levels are associated with individual differences in corticomuscular coherence during isometric voluntary contraction"

Ryosuke Matsuya[1], Junichi Ushiyama[2,3], and Junichi Ushiba[3,4*]

[1]Graduate School of Science and Technology, Keio University, Kanagawa, Japan;

[2]Faculty of Environment and Information Studies, Keio University, Kanagawa, Japan;

[3]Department of Rehabilitation Medicine, Keio University School of Medicine, Tokyo, Japan; [4]Department of Biosciences and Informatics, Faculty of Science and Technology, Keio University, Kanagawa, Japan.

***Corresponding author:**

Junichi Ushiba, Department of Biosciences and Informatics, Faculty of Science and Technology, Keio University, 3-14-1 Hiyoshi, Kouhoku-ku, Yokohama, Kanagawa 223-8522, Japan

Tel: +81-45-566-1678; Fax: +81-45-566-1678; Email: ushiba@brain.bio.keio.ac.jp

Appendix A. coherence function

Correlations between two signals ($\overline{\boldsymbol{C_{xy}(f)}}$) were determined by coherence using following equation:

$$\left|C_{xy}(f)\right| = \frac{\left|\overline{P_{xy}(f)}\right|^2}{\overline{P_{xx}(f)} \cdot \overline{P_{yy}(f)}} \qquad (1)$$

Where $\left|\overline{\boldsymbol{P_{xy}(f)}}\right|$ denotes the averaged cross-spectral density function for the signal *x* that is the EEG and *y* that is the rectified EMG throughout the segments at a given frequency *f*. $\overline{\boldsymbol{P_{xx}(f)}}$ and $\overline{\boldsymbol{P_{xy}(f)}}$ denote the averaged power-spectral density (PSD) functions for signal *x* and y throughout the segments at the same frequency. The coherence function evaluates a linear correlation on a scale of 0 to 1, where 1 represents a perfect linear correlation.

Appendix B. Significant level for CMC

To eliminate the false-positive, a Bonferroni correction across frequency bins between 3 and 50 Hz (including α, β, and γ-bands) was applied to the equation defining the significant level coherence (SL). Thus, when the confidence limit is α%, the SL is defined as follows:

$$SL(\alpha) = 1 - \left[\frac{1}{N} \cdot \left(1 - \frac{\alpha}{100}\right)\right]^{1/(L-1)} \qquad (\mathbf{2})$$

Where *N* is the number of frequency bins, and *L* is the number of epochs. In EXP_{ICI}, as an *N* of 80, *L* of 245, and α of 95 were chosen, the SL was determined to be 0.030. In EXP_{RI}, as an *N* of 40, *L* of 70, and α of 95 were chosen, the SL was determined to be 0.091.
